# Mechanical and oral antibiotics bowel preparation reduce the risk of surgical site infections and anastomotic leakage in colorectal surgery: a GRADE-based meta-analysis and trial sequential analysis

**DOI:** 10.3389/fmed.2026.1788204

**Published:** 2026-03-17

**Authors:** Kai Lu, Xuefeng Peng, Furui Zhong, Faqiang Zhang, Hua Yang, Ke Lan

**Affiliations:** Department of General Surgery, Zigong Fourth People‘s Hospital, Zigong, China

**Keywords:** bowel preparation, colorectal surgery, MBP, MOABP, surgical site infection

## Abstract

**Purpose:**

Recent updates from randomized controlled trials (RCTs) indicated that mechanical and oral antibiotics bowel preparation (MOABP) can reduce post-operative surgical site infection (SSI), but its effect on anastomotic leakage (AL) remains controversial. This study systematically reviews and conduct trial sequential analysis (TSA) of the RCTs to determine whether MOABP can reduce SSI and AL after colorectal surgery.

**Methods:**

We conducted a comprehensive search of PubMed, Cochrane Library, Embase, and Web of Science for RCTs comparing MOABP to mechanical bowel preparation (MBP) alone, from database inception to February 1, 2025. Study quality was assessed using the Cochrane Risk of Bias tool. Meta-analysis, subgroup analysis, and sensitivity analysis were conducted using RevMan 5.3.1 software. TSA was performed with TSA software to evaluate the robustness of the primary outcomes.

**Results:**

A total of 17 RCTs involving 4,633 patients were included. Meta-analysis showed that the MOABP significantly reduced SSI (OR = 0.44, 95% CI: 0.37–0.54, *p* < 0.00001, *I*^2^ = 33%, moderate-quality evidence) and AL (OR = 0.42, 95% CI: 0.30–0.57, *p* < 0.00001, *I*^2^ = 3%, moderate-quality evidence). Subgroup analyses indicated reductions in superficial SSI, deep SSI, space and organ infection. TSA further confirmed the robustness of these findings.

**Conclusion:**

MOABP can effectively reduce the risk of SSI and AL in colorectal surgery. Pre-operative bowel preparation should consider incorporating oral antibiotics to optimize surgical outcomes.

**Systematic Review Registration:**

https://www.crd.york.ac.uk/PROSPERO/recorddashboard.

## Background

Colorectal surgery represents a fundamental procedure in gastrointestinal operations, Yet post-operative surgical site infection (SSI) remain frequent complications, affecting approximately one-third of patients undergoing colorectal procedures (1). This clinical challenge persists despite colorectal cancer ranking as the second leading cause of cancer-related mortality worldwide, driving a substantial volume of colorectal resections annually (2, 3). Moreover, surgical intervention constitutes an essential treatment modality for refractory inflammatory bowel diseases. Current pre-operative protocols typically involve MBP utilizing oral laxatives or enema administration. Nevertheless, considerable controversy persists regarding the adjunctive use of pre-operative oral antibiotics, with no established consensus on optimal preparation regimens (4). Despite the ongoing debate, several guidelines have already recommended the use of oral antibiotics in combination with mechanical bowel preparation (5, 6). Recent years have witnessed renewed research interest in this domain, evidenced by multiple randomized controlled trials (RCTs) (7–13). Emerging evidence suggests that mechanical and oral antibiotics bowel preparation (MOABP) may confer superior SSI risk reduction compared to mechanical bowel preparation (MBP) monotherapy (7–9). However, contrary findings from a 2019 RCT failed to demonstrate significant benefits of MOABP (14), a conclusion subsequently corroborated by studies conducted by Lee H et al. (15) and Catarci M et al. (16). Similarly, contrasting evidence exists regarding its impact on anastomotic leakage (AL). While Koskenvuo L et al. (7) reported a protective association between MOABP and AL risk, subsequent investigations by Frountzas M et al. (8) and Lei P et al. (9) found no statistically significant differences. Notably, the current evidence base remains constrained by the absence of large-scale multicenter RCTs definitively establishing MOABP‘s efficacy in SSI and AL prevention following colorectal surgery. Given the Recent publication of several high-quality RCTs, we conducted this updated meta-analysis to comprehensively evaluate the therapeutic efficacy of MOABP and provide evidence-based guidance for clinical decision-making.

## Methods

### Literature search strategy

Two researchers independently conducted systematic searches in PubMed, Cochrane Library (Cochrane Central Register of Controlled Trials), Embase, and Web of Science databases, as well as other sources from their inception to February 1, 2025. The search strategy combined Medical Subject Headings (MeSH) terms and free-text words using Boolean operators “OR” and “AND”. Key search terms included: Inflammatory Bowel Disease, Colorectal Neoplasm^*^, Colorectal Tumor^*^, Colorectal Cancer^*^, Colorectal Carcinoma^*^, Rectum Neoplasm^*^, Rectal Tumor^*^, Rectal Cancer^*^, Rectum Cancer^*^, Rectal Neoplasm^*^, mechanical bowel preparation, and bowel preparation. The complete search strategy is provided in Supplementary material 1. Non-English publications were excluded. Additionally, we manually screened the reference lists of all included studies to identify any other potentially relevant publications. Following the PRISMA statement, we performed a meta-analysis of the retrieved literature, with all search processes and study selection procedures independently verified by both investigators to ensure accuracy.

### Inclusion and exclusion criteria

Inclusion criteria: the study eligibility was defined according to the PICOS framework: population: patients undergoing colorectal resection for colorectal neoplasms or inflammatory bowel disease. Intervention: mechanical and oral antibiotics bowel preparation. Comparison: mechanical bowel preparation alone. Outcomes: the primary outcome was surgical site infection. Secondary outcomes included anastomotic leakage rates and analyses of risk factors associated with SSI development. Only randomized controlled trials (RCTs) were included. Exclusion criteria: single-arm studies, review articles, case reports, letters to the editor, non-English publications, and studies with incomplete or inaccessible data were excluded. The eligibility assessment was independently performed by two investigators to ensure objectivity, with discrepancies resolved through consensus discussion.

### Quality assessment of included studies

The methodological quality of the RCTs was evaluated using the Cochrane Risk of Bias tool (RoB 2.0) for RCT. All domains were assessed, including random sequence generation, allocation concealment, blinding of participants and personnel, blinding of outcome assessment, incomplete outcome data, selective reporting, and other potential sources of bias. Two researchers independently performed the quality assessment. To ensure objectivity, discrepancies in evaluations were resolved through consultation with a third investigator until consensus was achieved. The risk of bias for each domain was categorized as “low,” “high,” or “some concerns” based on pre-defined criteria (Supplementary material 2). Subsequently, the overall risk of bias for each individual study was classified into three levels: (1) Low risk: the study was rated as low risk across all five domains; (2) Some concerns: the study was rated as raising some concerns in at least one domain, but with no domain rated as high risk; (3) High risk: the study was rated as high risk in at least one domain, or there were multiple domains with some concerns whose cumulative effect could significantly compromise the reliability of the results.

### Data extraction

All retrieved records were managed using Zotero reference management software. Two researchers independently performed a three-stage screening process: initial title and abstract review, full-text assessment, and final eligibility confirmation based on pre-defined inclusion and exclusion criteria. The following data were systematically extracted using standardized forms: i) Study characteristics: First author, publication year, country of origin, sample sizes of intervention and control groups. ii) Clinical outcomes: incidence of surgical site infections (categorized as superficial SSI, deep SSI, or organ/space Infection), AL. iii) Risk factor analysis: documented variables associated with post-operative infectious complications. All literature screening and data extraction were conducted independently by two researchers, who selected studies and extracted data according to the pre-defined inclusion and exclusion criteria. Any disagreements arising during the assessment were first resolved through discussion between the two researchers. If consensus could not be reached, a third senior researcher was consulted for arbitration.

### Study outcomes

The primary outcomes were the incidence of SSI, classified according to Centers for Disease Control and Prevention (CDC) criteria as superficial incisional, deep incisional, or organ/space infections. Secondary outcomes included AL rates and analyses of risk factors associated with SSI development.

### Statistical analysis

Data synthesis and statistical analyses were conducted using RevMan 5.3.1 (The Nordic Cochrane Center, The Cochrane Collaboration, Copenhagen) (17). For dichotomous outcomes, pooled effects were estimated as odds ratios (OR) with 95% confidence intervals (CI). Heterogeneity across studies was quantified using the *I*^2^ statistic. A random-effects model was applied when *I*^2^ exceeded 50% (indicating substantial heterogeneity), whereas a fixed-effects model was utilized for *I*^2^ values below 50%. The statistical significance threshold was pre-defined as α = 0.05. In scenarios with significant heterogeneity (*I*^2^ >50%), subgroup analyses were performed to explore potential sources of heterogeneity. Publication bias was assessed through visual inspection of funnel plots. Prior to evaluating risk factors for surgical site infections, raw data were logarithmically transformed to derive the log (OR) and corresponding standard error (SE). Final pooled effect estimates were reported as OR with 95% CI. The quality of evidence for the outcomes was assessed using the GRADEpro GDT software (version 3.6).

### Ethical statement

This meta-analysis utilized data from previously published studies, all of which had obtained ethical approval from their respective institutional review boards. As no new human or animal experiments were conducted, additional ethical approval was not required for this study. This study was prospectively registered in the PROSPERO international registry (Registration No. CRD420251008491).

### Trial sequential analysis

TSA was conducted using dedicated software (version 0.9.5.10 Beta) to evaluate the robustness of primary outcomes. The type I error rate was pre-defined at 0.01, and the statistical power was set to 90% (18). Event rates from control groups of eligible RCTs were extracted to calculate the pooled incidence of target outcomes in the control population. The relative risk reduction (RRR) was estimated under a low-bias assumption. Based on these parameters, the required sample size was computed to determine the minimum number of participants needed to achieve statistically conclusive results in the meta-analysis.

## Outcomes

### Study selection

**Figure 1:**
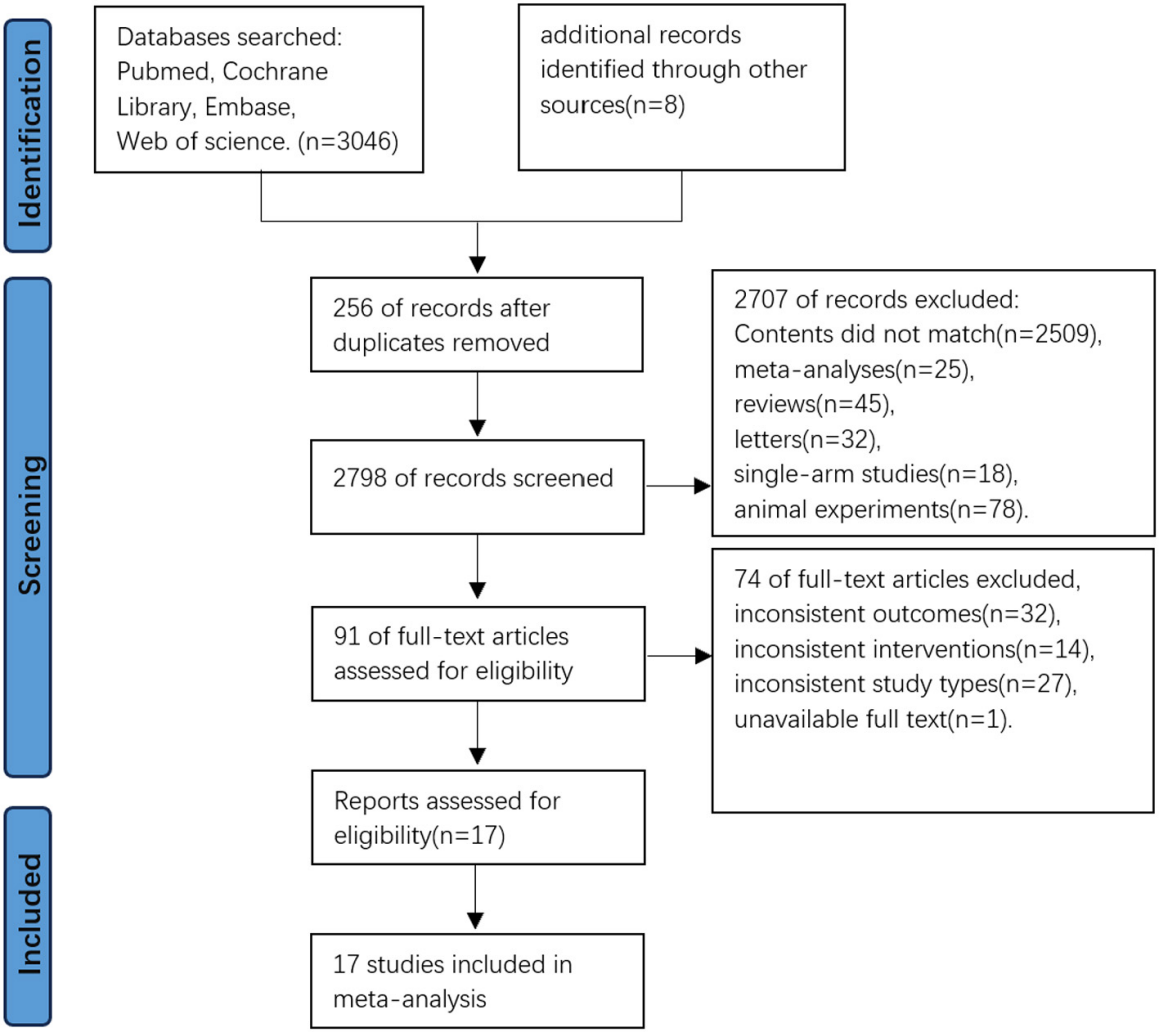
Flowchart of research screening process.

A total of 3,054 records were initially identified through database searches. After removing 256 duplicates, 2,798 studies underwent title and abstract screening. Of these, 2,678 were excluded for the following reasons: 2,389 irrelevant titles, 25 meta-analyses, 45 review articles, 32 letters/comments, 18 single-arm studies, and 78 animal experiments. Full-text evaluation of the remaining 120 articles led to the exclusion of 75 studies: 22 for mismatched outcomes, eight for inconsistent interventions, 27 for non-RCT designs, and one unavailable full text. Ultimately, 17 randomized controlled trials (RCTs) (7–13, 19–28) met all eligibility criteria and were included for quantitative synthesis (Figure 1).

### Study characteristics

Seventeen RCTs involving 4,633 patients were included in the meta-analysis, with 2,291 patients assigned to the MOABP group and 2,342 to the MBP group. Gender distribution was available for all studies except one by Clarke et al. (28), which did not report sex-specific data. Geographically, six studies were conducted in Japan, with seven high-quality RCTs published since 2020. Six studies included patients undergoing colorectal surgery without restriction on disease type. The study by Uchino M et al. (19) enrolled patients diagnosed with Crohn‘s disease, while the remaining studies included patients with colorectal tumors. All surgical procedures were elective. All studies compared SSI rates between groups, revealing significant disparities: SSI incidence ranged from 2 to 18.25% in the MOABP group vs. 7.5 to 43.27% in the MBP group. Subgroup analyses were performed in 12 studies, categorizing infections as superficial SSI, deep SSI, or organ/space infection, while 13 studies reported AL rates of 1.01 to 5.78% in the MOABP group and 2 to 13.56% in the MBP group. Six studies further identified MBP alone as an independent risk factor for SSI. Detailed characteristics of the included studies, including population demographics and outcome metrics, are summarized in Table 1 and Supplementary material 3.

**Table 1:** Baseline information for inclusion in the study.

**Author/year**	**Country**	Gender (M/F)		Age		Sample size		SSI		AL	
		**MOABP**	**MBP**	**MOABP**	**MBP**	**MOABP**	**MBP**	**MOABP**	**MBP**	**MOABP**	**MBP**
Laura Koskenvuo (7) 2024	Finland	158/119	190/98	70 (62–75)	69 (69–74)	277	288	23	48	16	39
Maximos Frountzas (8) 2024	Greece	118/85		701 ± 1		100	105	7	17	4	7
Purun Lei (9) 2023	China	102/55	95/57	62 (54, 70)	61 (54, 68)	157	152	13	27	7	14
Alberto Arezzo (10) 2021	Italy	53/47	56/48	70 (25, 95)		100	104	3	14	10	8
Evgeny Rybakov (11) 2021	Russia	24/33	31/28	65 (59; 66)	64 (59; 70)	57	59	2	13	2	8
G. Papp (12) 2021	Hungary	152/101	130/146	66.1 (12.1)	66.5 (12.3)	253	276	8	27	4	13
H. M. Schardey (13) 2020	Germany	28/12	24/16	64.1 (65)	64.58 (65)	40	40	1	3	2	8
MotoiUchino (19) 2019	Japan	115/48	128/34	38.51 ± 0.8	40.41 ± 3.4	126	126	23	27	/	/
Nadeem Anjum (20) 2017	China	61/34	59/36	46.3± 14.4	45.2± 15.6	95	95	7	26	/	/
A. Ikeda (21) 2016	Japan	141/114	141/115	65(27–93)	62 (29–86)	255	256	20	20	3	6
Hiroaki Hata (22) 2016	Japan	153/136	175/115	67(60.5–75)	67.5(60–75)	289	290	21	37	5	6
Sotaro Sadahiro (23) 2014	Japan	56/43	51/44	671 ± 1	661 ± 2	99	95	10	22	1	7
Minako Kobayashi (24) 2007	Japan	154/88	137/105	67.9 (31–92)	69.1 (46–95)	242	242	17	26	/	/
B.S. Reddy (25) 2007	United Kingdom	13/9	11/13	72.5(53–81)	68.5(61–75)	22	24	3	3	/	/
Hideyuki Ishida (26) 2001	Japan	47/25	42/29	62 (37–87)	65 (21–89)	72	71	8	17	1	2
D. M. Matheson (27) 1978	United Kingdom	28/23	35/24	64.7	63.2	51	59	9	25	0	7
James.Clarke (28) 1977	United States	/	/	61.3	63.5	56	60	5	21	1	7

MOABP, Mechanical and oral antibiotics bowel preparation; MBP, Mechanical bowel preparation; SSI, Surgical site infection; AL, Anastomotic leakage.

### Risk of bias assessment

**Figure 2:**
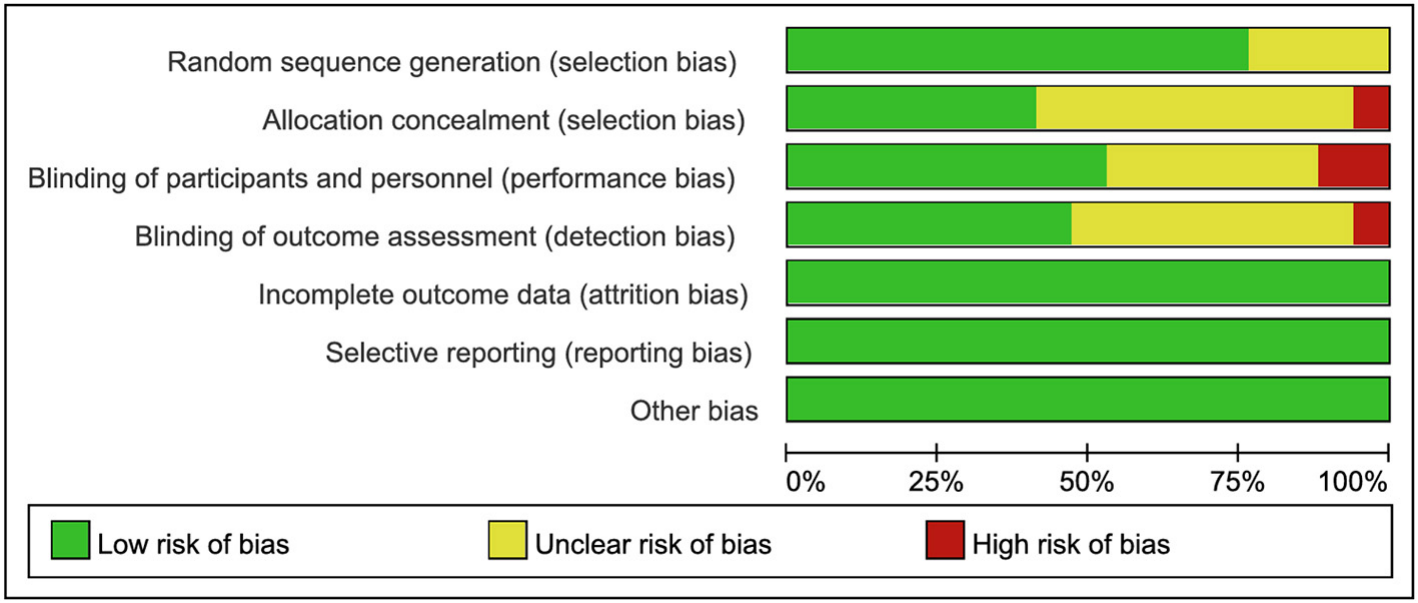
Risk of bias graph across included studies.

**Figure 3:**
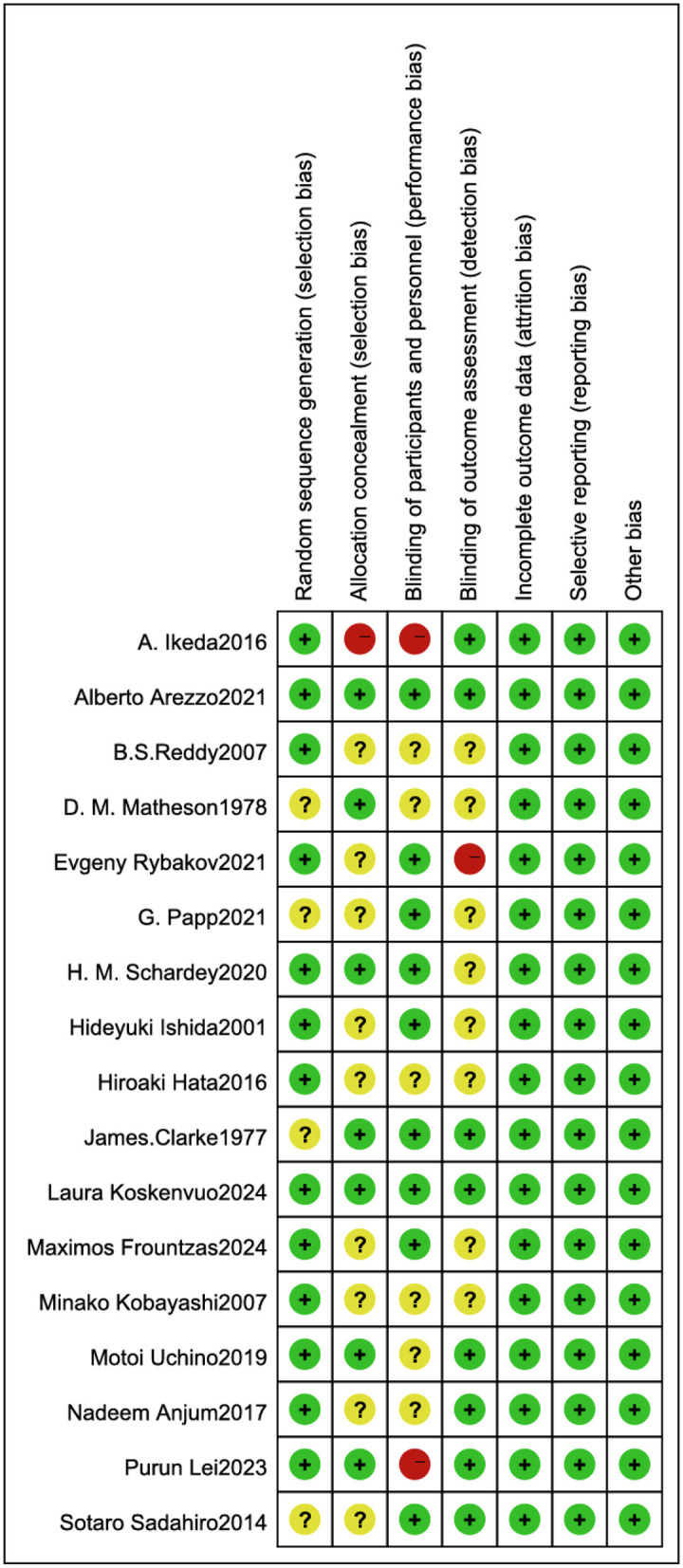
Risk of bias summary across included studies.

The methodological quality of the included RCTs was assessed using the Cochrane Risk of Bias tool. Three studies were identified as having high-risk biases: Ikeda A et al. (21) demonstrated high risk of performance bias due to unblinded investigators and participants, along with unclear allocation concealment; Rybakov E (11) exhibited high risk of detection bias from unblinded outcome assessors; and Lei P (9) showed high risk of performance bias with unblinded investigators and participants. Nine studies (8, 11, 12, 20, 22–26) lacked sufficient description of allocation concealment, while nine studies (8, 12, 13, 22, 24–27) did not report whether outcome assessors were blinded. The remaining studies maintained low-risk profiles across all domains. Overall, the included studies met criteria for low risk of bias, indicating acceptable methodological quality (Figures 2, 3).

### Assessment of evidence quality

**Figure 4:**
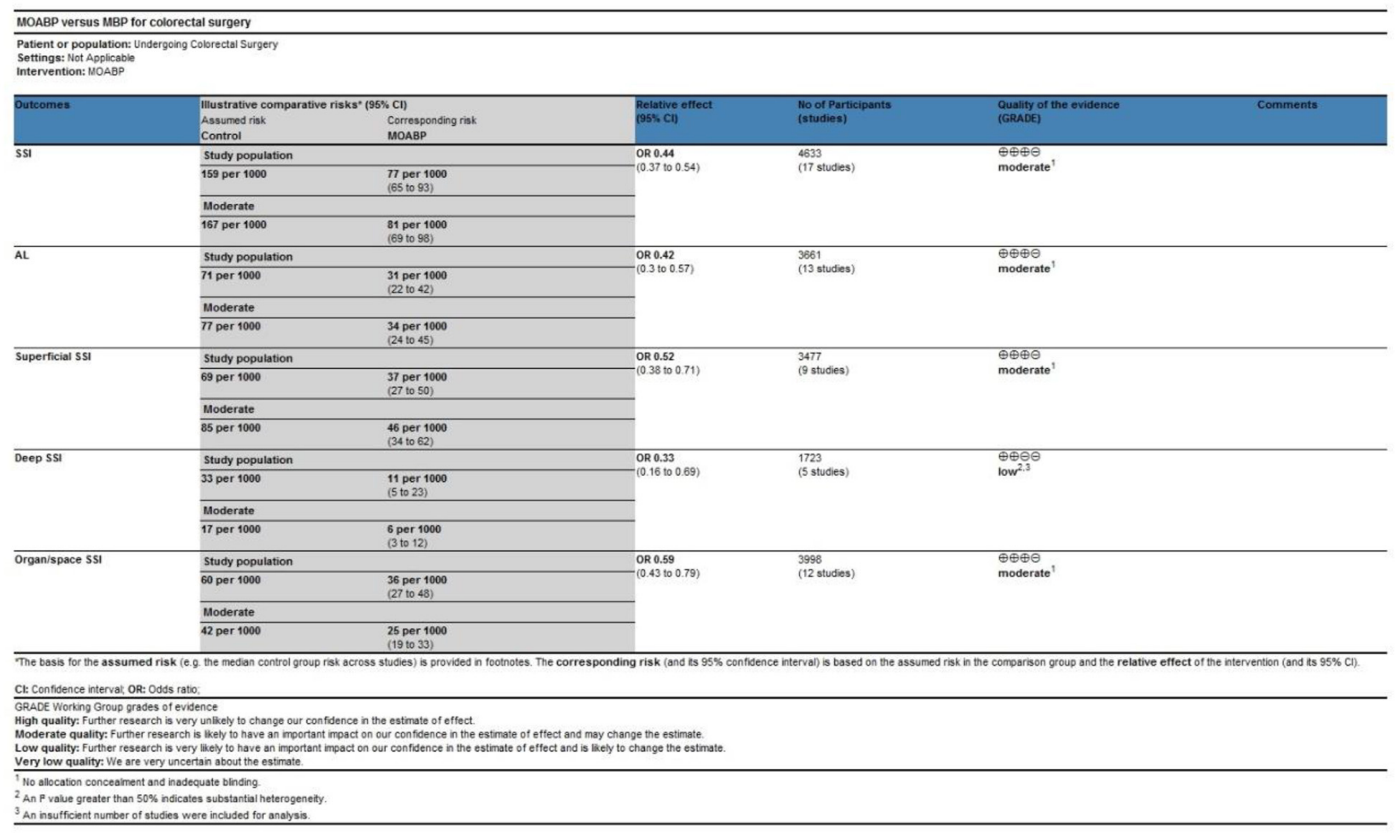
Assessment of the evidence level for study outcomes.

The certainty of evidence for the study outcomes was rated as moderate to low. Factors contributing to the downgrading of the evidence level included: lack of allocation concealment and inadequate blinding, an insufficient number of studies included in the analysis, and an *I*^2^ value greater than 50%, indicating substantial heterogeneity (Figure 4).

### Surgical site infection

**Figure 5:**
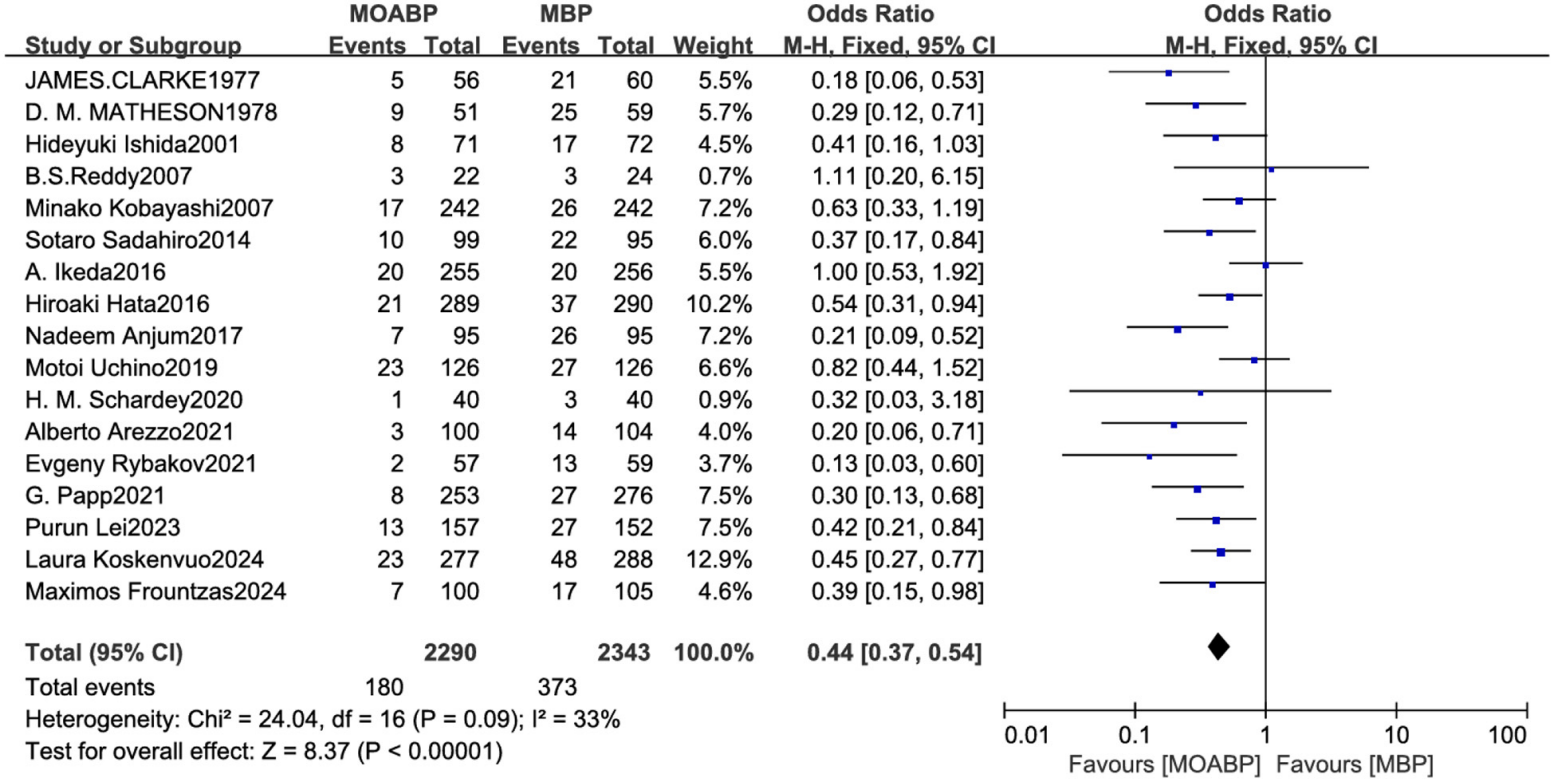
Forest plot of SSI comparing MOABP and MBP.

**Figure 6:**
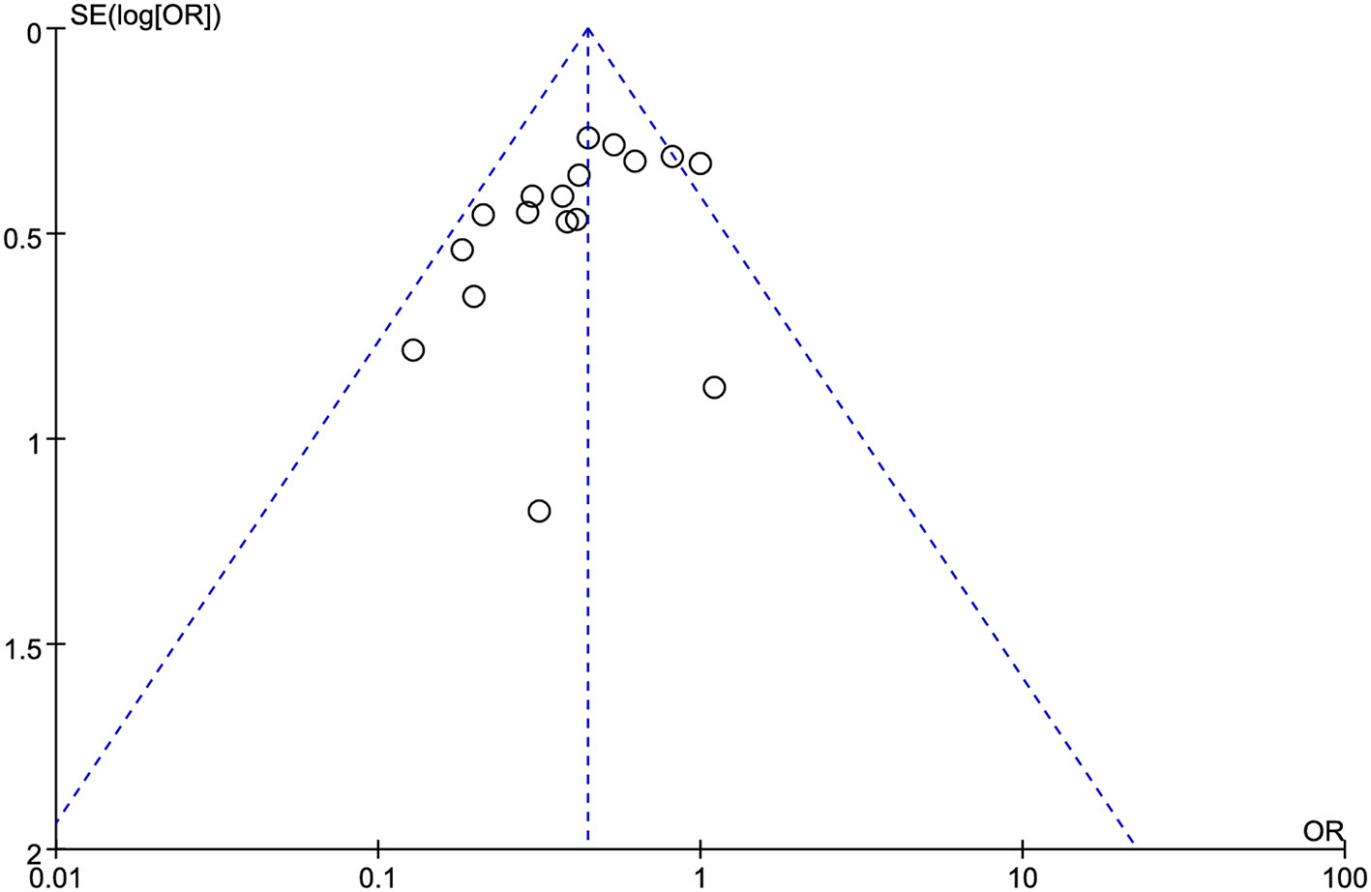
Funnel plot of SSI comparing MOABP and MBP.

Seventeen RCTs involving 4,633 patients (2,290 in the MOABP group and 2,343 in the MBP group) were analyzed. Post-operative SSI occurred in 180 (7.86%) patients in the MOABP group and 373 (15.91%) patients in the MBP group. Meta-analysis demonstrated a statistically significant reduction in SSI risk with MOABP (OR = 0.44, 95% CI: 0.37–0.54, *p* < 0.00001; *I*^2^ = 33%; Figure 5). Funnel plot analysis revealed symmetrical distribution of studies, indicating minimal publication bias (Figure 6).

### Anastomotic leakage

**Figure 7:**
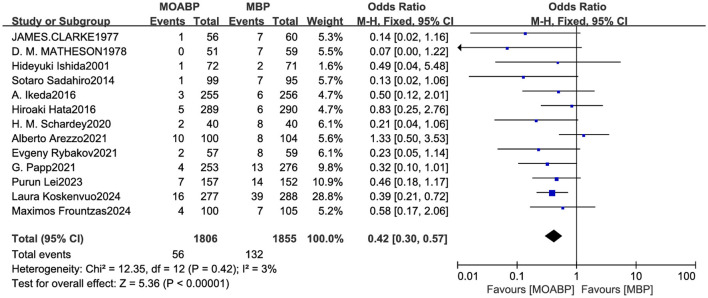
Forest plot of AL comparing MOABP and MBP.

**Figure 8:**
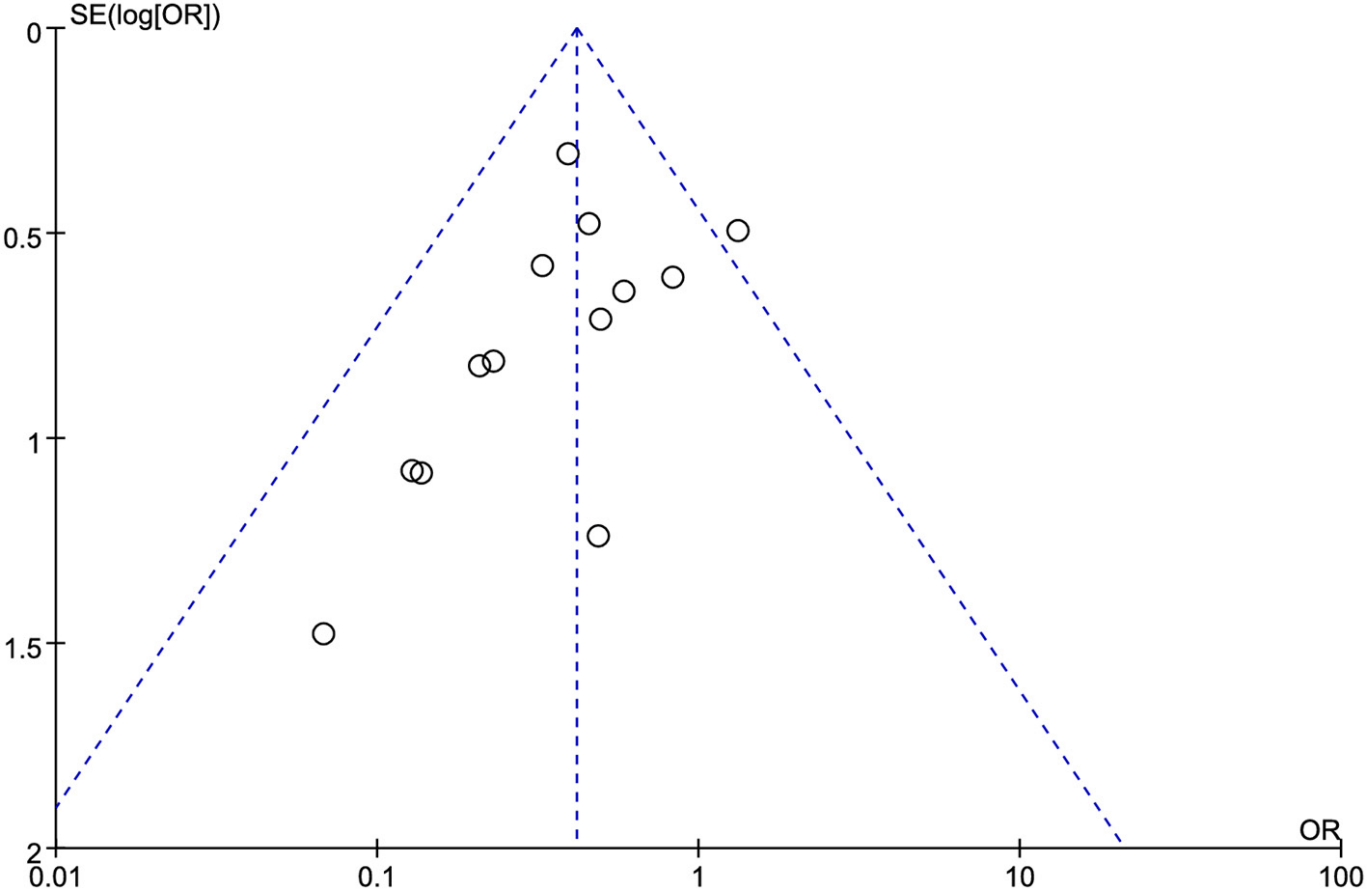
Funnel plot of AL comparing MOABP and MBP.

Thirteen studies involving 3,661 patients (1,806 in the MOABP group and 1,855 in the MBP group) systematically evaluated the incidence of AL following colorectal surgery. The analysis revealed 56 (3.1%) patients of anastomotic leakage in the MOABP group compared to 132 (7.1%) patients in the MBP group. Meta-analysis demonstrated a statistically significant reduction in AL rates favoring the MOABP protocol (OR = 0.42, 95% CI 0.30–0.57, *p* < 0.00001), with low heterogeneity across studies (*I*^2^ = 3%; Figure 7). Funnel plot evaluation showed symmetrical distribution of study outcomes, suggesting minimal publication bias in the included literature (Figure 8).

### Subgroup analysis

**Figure 9:**
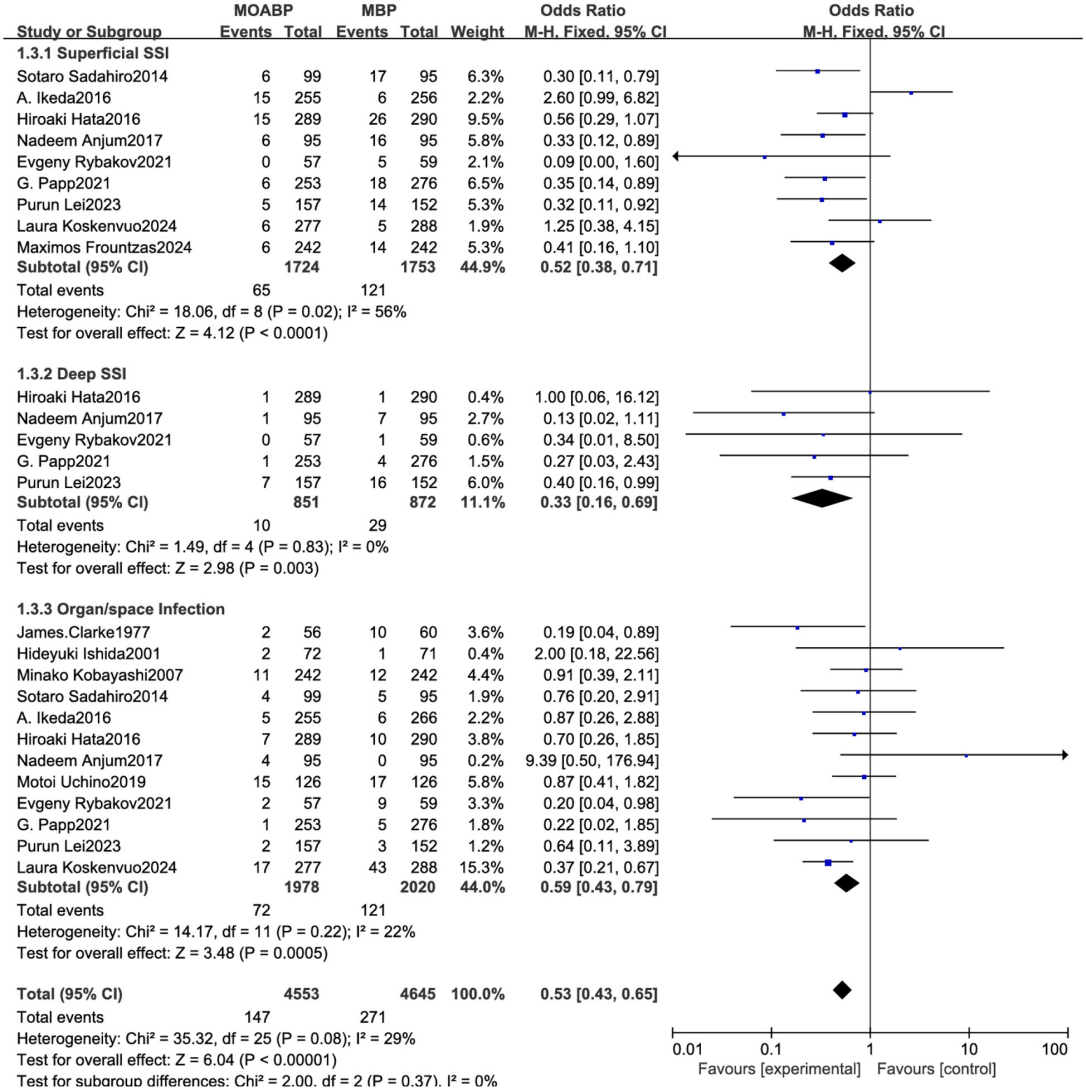
Forest plot of subgroup analysis for superficial SSI, Deep SSI and organ/space infection.

Superficial SSI: nine studies involving 3,477 patients (1,724 in the MOABP group and 1,753 in the MBP group) evaluated post-operative superficial SSI rates. Superficial incisional infection occurred in 65 (3.8%) patients in the MOABP group and 121 patients (6.9%) in the MBP group. Meta-analysis demonstrated a statistically significant difference between groups (OR = 0.52, 95% CI 0.38–0.71, *p* < 0.0001), though substantial heterogeneity was observed across studies (*I*^2^ = 56%). Sensitivity analysis using a leave-one-out approach identified the study by Ikeda A et al. (21) as the primary source of heterogeneity. After excluding this study, the pooled effect estimate remained statistically significant (OR = 0.42, 95% CI 0.30–0.59, *p* < 0.0001) with no residual heterogeneity (*I*^2^ = 0%), confirming the robustness of the findings. Deep SSI: five studies involving 1,723 patients (851 in the MOABP group and 872 in the MBP group) evaluated the incidence of deep SSI following colorectal surgery. Deep incisional infection occurred in 10 (1.2%) patients in the MOABP group compared to 29 (3.3%) patients in the MBP group. Meta-analysis revealed a statistically significant reduction in deep SSI rates favoring the MOABP protocol (OR = 0.33, 95% CI 0.16–0.69, *p* = 0.003), with no observed heterogeneity across studies (*I*^2^ = 0%). Organ/space Infection: 12 studies involving 3,998 patients (1,978 in the MOABP group and 2020 in the MBP group) was conducted to evaluate the incidence of organ/space infection. The analysis revealed 72 (3.64%) patients of organ/space infection in the MOABP group compared to 121 (5.99%) patients in the MBP group. A statistically significant difference in organ/space Infection was observed between the two groups (OR = 0.59, 95% CI 0.43–0.79, *p* = 0.0005), with low heterogeneity (*I*^2^ = 22%) across studies. These findings suggest that MOABP demonstrates superior efficacy in reducing organ/space infection risk compared to MBP (Figure 9).

### Risk factors for SSI

**Figure 10:**
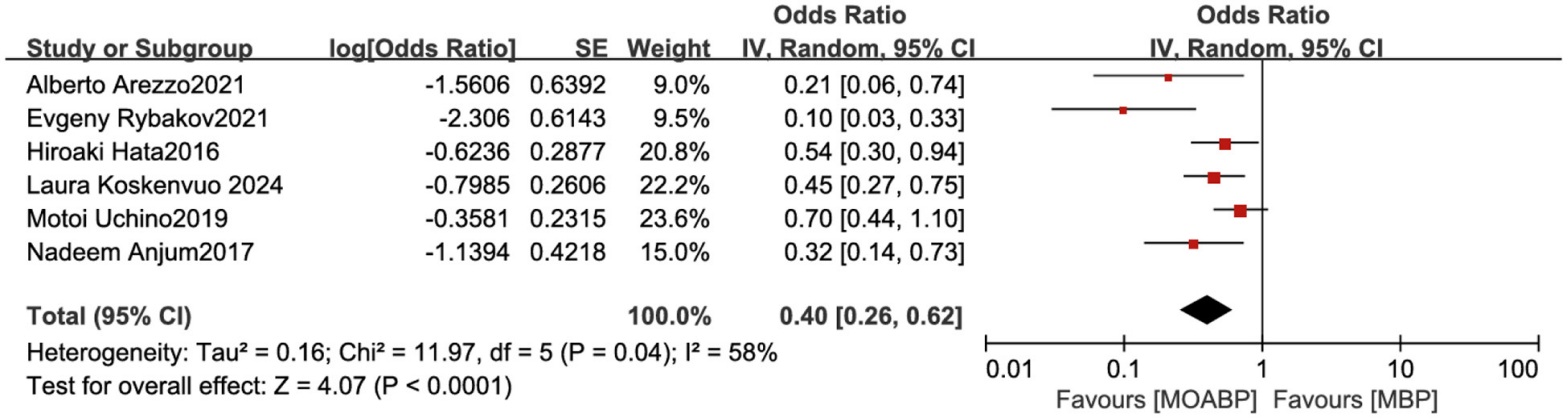
Forest plot of risk factor analysis for surgical site infection.

Six studies investigated risk factors for SSI, with meta-analysis demonstrating that MBP alone served as a risk factor for SSI. In contrast, the MOABP significantly reduced infection rates (OR = 0.40, 95% CI 0.26–0.62, *p* < 0.0001), though moderate heterogeneity was observed across studies (*I*^2^ = 58%; Figure 10). Sensitivity analysis using a leave-one-out approach identified the study by Rybakov E et al. (11) as the primary source of heterogeneity. Exclusion of this study yielded a consistent effect estimate (OR = 0.49, 95% CI 0.36–0.68, *p* < 0.0001) with substantially reduced heterogeneity (*I*^2^ = 24%), confirming the robustness of the association.

### Trial sequential analysis

**Figure 11:**
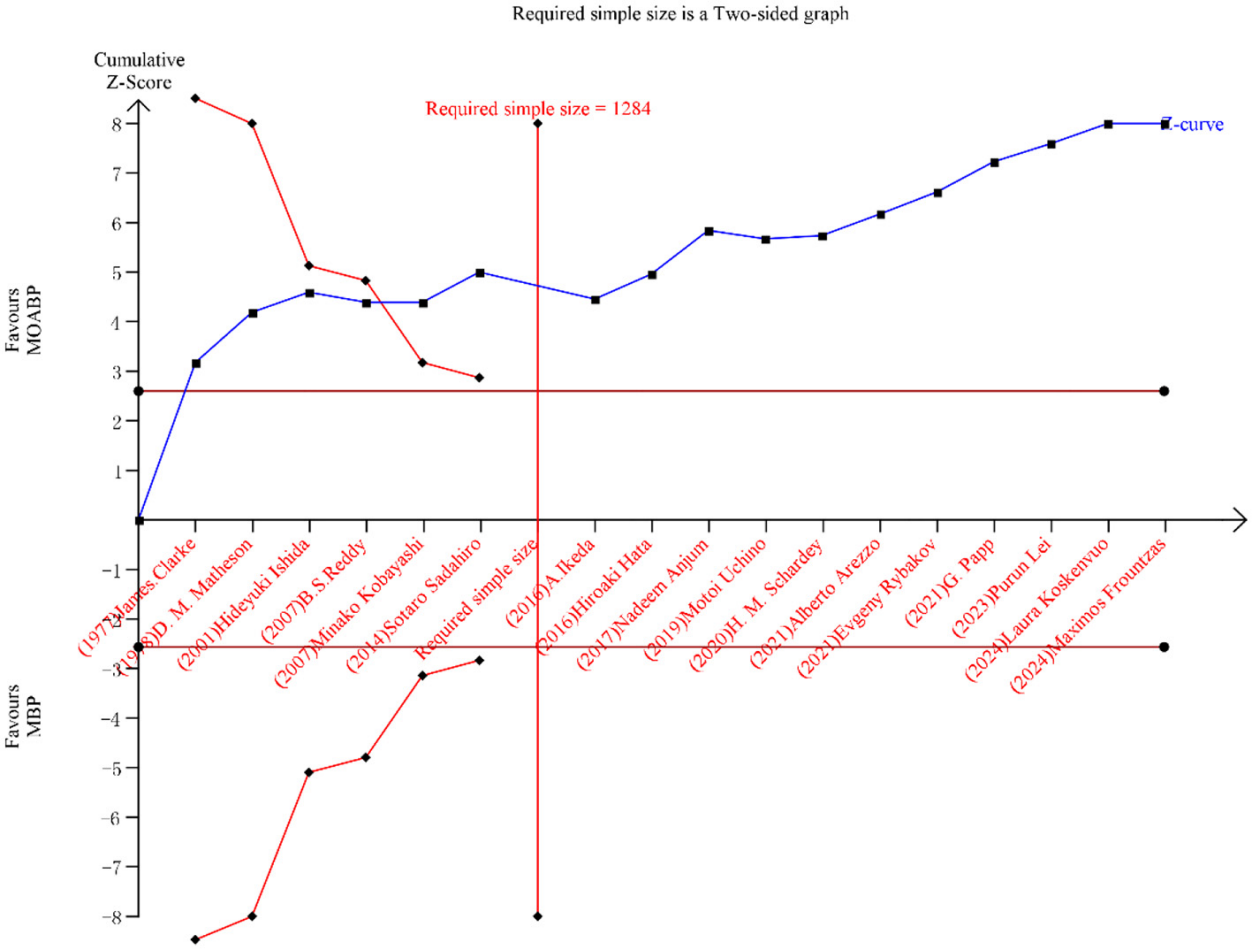
Trial sequential analysis for SSI.

**Figure 12:**
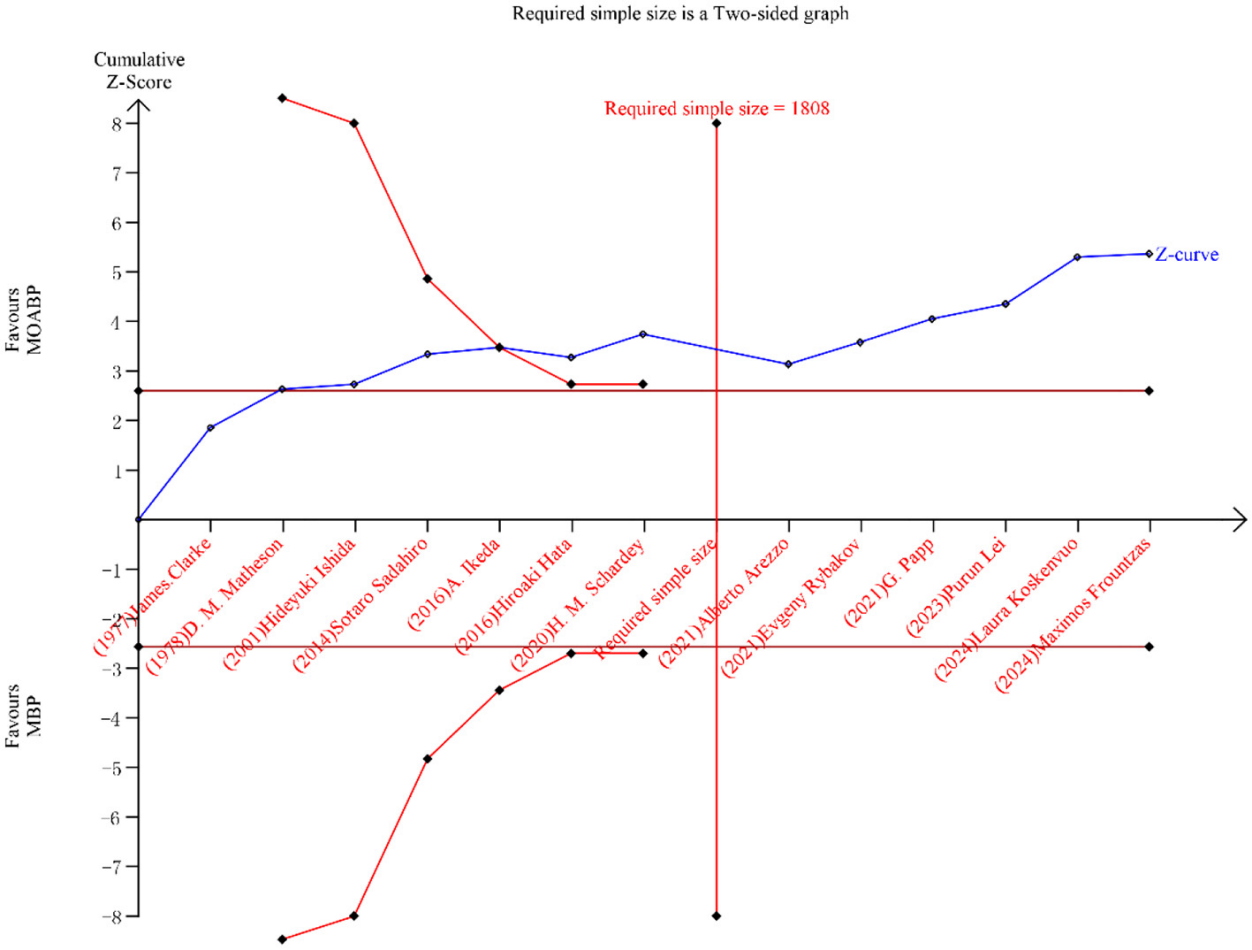
Trial sequential analysis for AL.

TSA was conducted to assess the conclusiveness of evidence for SSI and AL. The cumulative Z-curves for both outcomes crossed the pre-defined traditional significance boundaries, exceeded the TSA-adjusted monitoring thresholds, and surpassed the required sample size (Figures 11, 12). This indicates robust evidence with a low probability of type I or II errors, confirming that further studies are unlikely to alter the established conclusions.

## Discussion

SSI following colorectal procedures remain a significant clinical concern, imposing substantial economic and psychological burdens on patients (29). A Japanese study reported an SSI rate of approximately 10% after colorectal surgery (30), while a National Surgical Quality Improvement Program analysis demonstrated temporal trends: superficial and deep incisional infection rates declined from 5.9 and 3.3% in 2013 to 1.4 and 0.6% in 2020, respectively, though organ/space infection increased from 5.2 to 7.1% during this period (31). Recent evidence indicates that risk factors for SSI after colorectal surgery have expanded beyond traditional patient demographics to include more modifiable perioperative variables. In addition to well-established factors such as diabetes and obesity, pre-operative nutritional status and hypoalbuminemia have also been confirmed to be significantly associated with SSI (32–34). To mitigate SSI risks, the World Health Organization endorses antibiotic-enhanced bowel preparation protocols (35). Recent RCT have further demonstrated that oral antibiotics do not increase the risk of gut microbiota dysbiosis, with only one documented case of Clostridium difficile infection across these studies (7). However, current practice in many regions still prioritizes MBP alone, with ongoing debate regarding the additive benefit of oral antibiotics. A large-scale Korean retrospective study controversially identified oral antibiotics as a protective factor against SSI while concluding no significant overall effect (36). In contrast, investigations by Hansen RB et al. (37) and Yue Y et al. (38) demonstrated reduced SSI risks with MOABP, though these studies lacked granular subgroup analyses of superficial and deep SSI, organ/space infection, and associated risk factors. Notably, multiple RCTs are currently underway to further evaluate MOABP‘s efficacy (39, 40). This meta-analysis synthesizes evidence from 17 high-quality RCTs, including recently published trials, to systematically assess MOABP‘s impact. Our findings robustly support MOABP‘s superiority over MBP in reducing overall SSI rates after colorectal surgery. Subgroup analyses further revealed consistent benefits across infection subtypes: superficial SSI (OR = 0.52, 95% CI 0.38–0.71), deep SSI (OR = 0.33, 95% CI 0.16–0.69), and organ/space infections (OR = 0.42, 95% CI 0.30–0.57), all with statistical significance (*p* < 0.01). Crucially, our risk factor analysis confirmed MBP alone as a risk factor of SSI (OR = 0.40, 95% CI 0.26–0.62, *p* < 0.0001), underscoring the clinical imperative for antibiotic augmentation in bowel preparation protocols.

AL remains a frequent and severe complication following colorectal surgery, with reported incidence rates ranging from 3 to 24% (41–43). AL is associated with prolonged hospitalization, increased healthcare costs, reoperation risks, and elevated mortality. The role of pre-operative MOABP in mitigating AL remains contentious. While a retrospective study suggested MOABP reduces AL risk (44), and a prior meta-analysis reported its efficacy specifically for AL without impacting other complications (45), recent RCTs have yielded conflicting conclusions. Koskenvuo L et al. (7) observed reduced AL rates with MOABP, whereas studies by Frountzas M et al. (6), Lei P et al. (9), and Catarci M's propensity-matched analysis (46) demonstrated no significant differences. Our meta-analysis, synthesizing 13 RCTs involving 3,661 patients, identified 59 AL cases in the MOABP group vs. 136 in the MBP group, revealing a statistically significant risk reduction favoring MOABP (OR = 0.42, 95% CI 0.31–0.58, *p* < 0.0001, *I*^2^ = 5%). However, a critical limitation persists: none of the included studies stratified outcomes by anastomotic location, precluding subgroup analyses to evaluate site-specific AL risks. Given the well-documented anatomical and physiological differences between colonic and rectal anastomoses, future studies must systematically address this variable to refine pre-operative optimization strategies.

Trial sequential analysis, a methodological advancement addressing the inherent limitations of conventional meta-analyses (47, 48), was employed to validate the robustness of our findings. For both SSI and AL, the cumulative Z-curves surpassed three critical thresholds: i) traditional statistical significance, ii) TSA-adjusted monitoring boundaries, and iii) the required sample size. This tripartite confirmation indicates conclusive evidence with a < 5% probability of false-positive or false-negative conclusions, thereby solidifying the reliability of MOABP‘s protective effects. Notably, while our analysis demonstrates MOABP‘s efficacy in reducing AL risk (*p* < 0.0001), the current evidence base remains constrained by the absence of large-scale, multicenter RCT explicitly designed to evaluate anastomotic outcomes. Future studies must prioritize standardized protocols for anastomotic location documentation and intraoperative risk stratification to resolve lingering uncertainties.

Study limitations: first, inadequate allocation concealment and blinding in some of the included studies may have led to an overestimation of the effect size of MOABP. Second, considerable heterogeneity was observed among the studies in relation to superficial SSI, which could affect the stability of the pooled results. Moreover, inconsistencies in the oral antibiotic regimens used in the MOABP groups across the studies may limit the generalizability and clinical applicability of the findings. Finally, the lack of blinding in the study selection process may have introduced subjective bias, potentially reducing the reliability of the results.

## Conclusion

MOABP significantly reduces the risk of SSI and AL following colorectal surgery. The inclusion of oral antibiotics in pre-operative bowel preparation is strongly recommended. Incorporating oral antibiotics into pre-operative bowel preparation may be a beneficial strategy.

## Data Availability

The original contributions presented in the study are included in the article/Supplementary material, further inquiries can be directed to the corresponding author.
