# Supplementary material for: Mechanical and oral antibiotics bowel preparation reduce the risk of surgical site infections and anastomotic leakage in colorectal surgery: a GRADE-based meta-analysis and trial sequential analysis
Source: Front Med (Lausanne). 2026 Mar 17;13:1788204. doi: 10.3389/fmed.2026.1788204 (PMC13035496; doi:10.3389/fmed.2026.1788204)
Supplement: Supplementary file 1 [file Table_1.docx]

**Pubmed**

Inflammatory Bowel Diseases"[Mesh] OR "Colorectal Neoplasms"[Mesh] OR "Colonic Neoplasms"[Mesh] OR "Rectal Neoplasms"[Mesh] OR inflammatory bowel disease*[tiab] OR IBD[tiab] OR Crohn*[tiab] OR "Ulcerative Colitis"[tiab] OR "Colorectal Neoplasm*"[tiab] OR "Colorectal Tumor*"[tiab] OR "Colorectal Cancer*"[tiab] OR "Colorectal Carcinoma*"[tiab] OR "Colon Neoplasm*"[tiab] OR "Colon Cancer*"[tiab] OR "Rectum Neoplasm*"[tiab] OR "Rectal Tumor*"[tiab] OR "Rectal Cancer*"[tiab] OR "Rectal Carcinoma*"[tiab]) AND ("Preoperative Care"[Mesh] OR mechanical bowel preparation[tiab] OR bowel preparation[tiab] OR MBP[tiab] OR intestinal preparation[tiab]) AND (randomized controlled trial[pt] OR controlled clinical trial[pt] OR randomized[tiab] OR randomised[tiab] OR placebo[tiab] OR clinical trials as topic[mh] OR randomly[tiab] OR trial[ti])

**Embase:**

1. exp inflammatory bowel disease/ or exp crohn disease/ or exp ulcerative colitis/

2. exp colorectal cancer/

3. (inflammatory bowel disease* or IBD or Crohn* or "Ulcerative Colitis").ti,ab,kw.

4. (Colorectal Neoplasm* or Colorectal Cancer* or Colon Cancer* or Rectal Cancer*).ti,ab,kw.

5. 1 or 2 or 3 or 4

6. (mechanical bowel preparation or bowel preparation or MBP or "bowel prep").ti,ab,kw.

7. 5 and 6

8. exp randomized controlled trial/

9. random*.ti,ab.

10. trial.ti.

11. 8 or 9 or 10

12. 7 and 11

**Cochrane Library**

(("Inflammatory Bowel Diseases" OR "Colorectal Neoplasms" OR inflammatory bowel disease* OR IBD OR Crohn* OR "Ulcerative Colitis" OR Colorectal Neoplasm* OR Colorectal Cancer* OR Colon Cancer* OR Rectal Cancer*):ti,ab,kw) AND ((mechanical bowel preparation OR bowel preparation OR MBP OR "bowel prep"):ti,ab,kw)

**Web of Science**

TS=(("Inflammatory Bowel Disease*" OR IBD OR Crohn* OR "Ulcerative Colitis" OR "Colorectal Neoplasm*" OR "Colorectal Cancer*" OR "Colon Cancer*" OR "Rectal Cancer*")) AND TS=("mechanical bowel preparation" OR "bowel preparation" OR MBP OR "bowel prep")
