# Supplementary material for: Mechanical and oral antibiotics bowel preparation reduce the risk of surgical site infections and anastomotic leakage in colorectal surgery: a GRADE-based meta-analysis and trial sequential analysis
Source: Front Med (Lausanne). 2026 Mar 17;13:1788204. doi: 10.3389/fmed.2026.1788204 (PMC13035496; doi:10.3389/fmed.2026.1788204)
Supplement: Supplementary file 2 [file Table_2.docx]

| Domain | Risk level | Criteria |
| --- | --- | --- |
| Random sequence generation | Low risk | The investigators describe a random component in the sequence generation process (e.g., random number table, computer random number generator, coin tossing, shuffling cards or envelopes) |
|  | Some concerns | The study is described as randomized but no detailed description of the sequence generation method is provided |
|  | High risk | A non-random method is used for sequence generation (e.g., odd or even date of birth, hospital or clinic record number, alternation, or judgment of the clinician) |
| Allocation concealment | Low risk | Central allocation (e.g., telephone, web-based, or pharmacy-controlled randomization) or sequentially numbered, opaque, sealed envelopes were used to conceal allocation |
|  | Some concerns | The method of allocation concealment is not clearly described, but there is no indication that it was inadequate |
|  | High risk | Allocation was based on an open random allocation schedule (e.g., a list of random numbers); or envelopes were unsealed, non-opaque, or not sequentially numbered; or alternation or rotation was used |
| Blinding of participants and personnel | Low risk | Blinding of participants and key study personnel ensured, and unlikely that the blinding could have been broken; or no blinding or incomplete blinding, but the outcome is not likely to be influenced by lack of blinding (e.g., objective outcomes such as mortality) |
|  | Some concerns | Blinding stated but not described, or no blinding but outcome measurement unlikely to be influenced, though some uncertainty remains |
|  | High risk | No blinding or incomplete blinding, and the outcome is likely to be influenced by lack of blinding; or blinding attempted but likely broken |
| Blinding of outcome assessment | Low risk | Blinding of outcome assessors ensured, and unlikely that the blinding could have been broken; or no blinding of outcome assessment, but the outcome measurement is not likely to be influenced by lack of blinding (e.g., objective outcomes) |
|  | Some concerns | Blinding of outcome assessment stated but not described, or no blinding but outcome measurement unlikely to be influenced, though some uncertainty remains |
|  | High risk | No blinding of outcome assessment, and the outcome measurement is likely to be influenced by lack of blinding; or blinding attempted but likely broken |
| Incomplete outcome data | Low risk | No missing outcome data; or reasons for missing outcome data unlikely to be related to true outcome; or missing outcome data balanced in numbers across groups, with similar reasons; or missing data have been imputed using appropriate methods |
|  | Some concerns | Proportion of missing outcomes is moderate (5-20%) and reasons may be related to outcome, but sensitivity analyses show robustness; or missing data not balanced across groups |
|  | High risk | Reason for missing outcome data likely to be related to true outcome, with either imbalance in numbers or reasons for missing data across groups; or "as-treated" analysis done with substantial departure from allocation; or potentially inappropriate application of imputation |
| Selective reporting | Low risk | The study protocol is available and all of the study's pre-specified (primary and secondary) outcomes have been reported in the pre-specified way; or no protocol but all expected outcomes (including relevant negative findings) are reported |
|  | Some concerns | No study protocol is available, but all expected outcomes are reported; or insufficient information to judge |
|  | High risk | Not all of the study's pre-specified primary outcomes have been reported; or one or more primary outcomes are reported using measurements, analysis methods, or subsets of data that were not pre-specified; or one or more reported primary outcomes were not pre-specified |
| Other potential sources of bias | Low risk | The study appears to be free of other sources of bias (e.g., no extreme baseline imbalance, no early stopping for benefit, no funding or conflicts likely to bias results) |
|  | Some concerns | There may be a risk of bias, but there is insufficient information to assess whether an important risk of bias exists; or the available information suggests a potential bias that could raise concerns |
|  | High risk | There is at least one important risk of bias not covered by other domains (e.g., extreme baseline imbalance, fraudulent behavior, inappropriate study design, or significant conflicts of interest) |

supplementary material 2. Risk of bias assessment criteria using the Cochrane RoB 2.0 tool
