# Supplementary material for: Mechanical and oral antibiotics bowel preparation reduce the risk of surgical site infections and anastomotic leakage in colorectal surgery: a GRADE-based meta-analysis and trial sequential analysis
Source: Front Med (Lausanne). 2026 Mar 17;13:1788204. doi: 10.3389/fmed.2026.1788204 (PMC13035496; doi:10.3389/fmed.2026.1788204)
Supplement: Supplementary file 3 [file Table_3.docx]

| Author/year | Bowel preparation regimen | | Diabetes | | Hypertension | |
| --- | --- | --- | --- | --- | --- | --- |
|  | MOABP | MBP | MOABP | MBP | MOABP | MBP |
| Laura Koskenvuo^7^ 2024 | 1g of neomycin and 1g of metronidazole | polyethylene glycol solution | 48 | 38 | 114 | 93 |
| MaximosFrountzas^8^ 2024 | three doses of 400 mg rifaximin and a single dose of 500 mg metronidazole | two doses of orally administered sodium phosphate solution | 42 | | 100 | |
| Purun Lei^9^ 2023 | streptomycin 1 g and metronidazole 0.2 g | polyethylene glycol with 2 L water | 38 | 36 | 46 | 44 |
| Alberto Arezzo^10^ 2021 | neomycin 25,000 UI and Bacitracin 2500 UI | according to local habits | 17 | 14 | / | / |
| Evgeny Rybakov^11^ 2021 | erythromycin 500 mg and metronidazole 500 mg | polyethylene glycol solution | 10 | 3 | / | / |
| G. Papp^12^ 2021 | 500 mg metronidazole and 1000 mg eomycin sulphate | 40 ml castor oil with 20 mL paraffin | 44 | 50 | / | / |
| H. M. Schardey^13^ 2020 | polymyxin B (100 mg), tobramycin (80 mg), and vancomycin (125 mg) | 3–6 L of colonoscopy solution . | / | / | / | / |
| MotoiUchino^19^ 2019 | 500mg of kanamycin and 500mg of metronidazole | 20mL of 0.75% sodium picosulfate hydrate magnesium citrate solution | 1 | 2 | / | / |
| Nadeem Anjum^20^ 2017 | metronidazole 400 mg and levofloxacin 200 mg orally | sodium phosphate 133 mL | 3 | 2 | / | / |
| A. Ikeda^21^ 2016 | 750mg metronidazole with 1000mg kanamycin | magnesium cit rate and sodium picosulfate | 37 | 31 | / | / |
| Hiroaki Hata^22^ 2016 | 1 g of kanamycin and 750 mg of metronidazole | 75 mg of sodium picosulfate and 34 g of magnesium citrate | 33 | 31 | / | / |
| Sotaro Sadahiro^23^ 2014 | 0.5 g kanamycin sulfate and 0.5g of metronidazole | 10 mL of sodium picosulfate and 2,000 mL of polyethylene glycol–electrolyte sodium | / | / | / | / |
| MinakoKobayashi^24^ 2007 | 1 g kanamycin and 400 mg erythromycin | polyethylene glycol | / | / | / | / |
| B.S. Reddy^25^ 2007 | 3 g oral neomycin in three divided doses | sodium picosulphate and magnesium citrate | / | / | / | / |
| Hideyuki Ishida^26^ 2001 | kanamycin 2g/day and erythromycin 1.6g/day for 2 days | 2L polyethylene glycol lavage performed | 7 | 6 | 10 | 10 |
| D. M. Matheson^27^ 1978 | neomycin 1 g and metronidazole 200 mg | five 15 g doses of oral magnesium sulphate 2 days |  |  |  |  |
| James.Clarke^28^ 1977 | 1g neomycin sulfate and 1g erythromycin base | Magnesium sulfate 30 mL | 5 | 4 | / | / |

Supplementary material 3: MOABP: Mechanical and oral antibiotics bowel preparation; MBP: Mechanical bowel preparation
